# Can 3D Printed Tablets Be Bioequivalent and How to Test It: A PBPK Model Based Virtual Bioequivalence Study for Ropinirole Modified Release Tablets

**DOI:** 10.3390/pharmaceutics16020259

**Published:** 2024-02-09

**Authors:** Olha Shuklinova, Gabriela Wyszogrodzka-Gaweł, Ewelina Baran, Bartosz Lisowski, Barbara Wiśniowska, Przemysław Dorożyński, Piotr Kulinowski, Sebastian Polak

**Affiliations:** 1 Doctoral School of Medical and Health Sciences, Jagiellonian University Medical College, 16 Łazarza St., 31-530 Kraków, Poland; 2 Simcyp Division, Certara UK Limited, Level 2-Acero, 1 Concourse Way, Sheffield S1 2BJ, UK; sebastian.polak@uj.edu.pl; 3 Faculty of Pharmacy, Jagiellonian University Medical College, Medyczna 9, 30-688 Krakow, Poland; gabriela.wyszogrodzka@uj.edu.pl (G.W.-G.); bartek.lisowski@uj.edu.pl (B.L.); b.wisniowska@uj.edu.pl (B.W.); przemyslaw.dorozynski@uj.edu.pl (P.D.); 4 Institute of Technology, University of the National Education Commission, Podchorążych 2, 30-084 Kraków, Poland; ewelina.baran@up.krakow.pl (E.B.); piotr.kulinowski@up.krakow.pl (P.K.)

**Keywords:** ropinirole, virtual bioequivalence, physiologically based pharmacokinetic modeling, 3D printing

## Abstract

As the field of personalized dosing develops, the pharmaceutical manufacturing industry needs to offer flexibility in terms of tailoring the drug release and strength to the individual patient’s needs. One of the promising tools which have such capacity is 3D printing technology. However, manufacturing small batches of drugs for each patient might lead to huge test burden, including the need to conduct bioequivalence trials of formulations to support the change of equipment or strength. In this paper we demonstrate how to use 3D printing in conjunction with virtual bioequivalence trials based on physiologically based pharmacokinetic (PBPK) modeling. For this purpose, we developed 3D printed ropinirole formulations and tested their bioequivalence with the reference product Polpix. The Simcyp simulator and previously developed ropinirole PBPK model were used for the clinical trial simulations. The Weibull-fitted dissolution profiles of test and reference formulations were used as inputs for the model. The virtual bioequivalence trials were run using parallel design. The study power of 80% was reached using 125 individuals. The study demonstrated how to use PBPK modeling in conjunction with 3D printing to test the virtual bioequivalence of newly developed formulations. This virtual experiment demonstrated the bioequivalence of one of the newly developed formulations with a reference product available on a market.

## 1. Introduction

While most of the drug products are still dosed using the ‘one dose fits all’ principle, there is a growing number of scientific reports which indicate the importance of accounting for the interindividual variability in physiological parameters governing pharmacokinetics and pharmacodynamics of the drug in the population [1,2,3]. The term ‘personalized dosing’ usually refers to the prediction of the treatment regimen for a particular patient, considering information about the patient’s physiology, demographics, drug substance properties, and the formulation properties [4]. In some diseases, the treatment regimens are adjusted dynamically during the treatment to improve the patient’s pharmacodynamic response or decrease the unwanted drug effects. For the illustration of such a case, we would like to refer to the ropinirole dosing in Parkinson’s disease. The ropinirole-specific dopaminergic side effects do not allow the target effective dose to be administered at the beginning of treatment. The patient must go through the long and complicated titration period where the dose is increased slowly to the target effective dose. At the same time, the ropinirole release from the formulation has to be smooth to mimic physiological stimulation of the dopamine receptors as close as possible, which suggests that the modified release formulation will be more beneficial compared to the immediate release. The approved strengths of modified release formulations include 2, 4, and 8 mg, which indicates that the dose increase can only be conducted with the step that is not smaller than 2 mg; therefore, clinicians do not have a possibility to use smaller steps such as 1 mg or 0.5 mg for this formulation. In such cases, ‘personalization’ can be extended to the manufacturing level, which can also be performed on an individual basis using the three-dimensional (3D) printing technologies. The 3D printing can offer flexibility in manufacturing individualized solid oral dosage forms with customizable doses (for example, 1, 6, or 5.5 mg of the above mentioned ropinirole), release profiles, and the exact number of tablets the patient needs [5,6].

However, before being implemented into routine practice, 3D printing technologies have to overcome technological challenges such as a quality control testing strategy for small batches [7,8]. Additionally, the question of bioequivalence (BE) of newly printed formulations arises. Currently, the bioequivalence study with the pharmacokinetic endpoints is one of the main studies for the orally taken drug. It is performed to not only compare innovative and generic drug products, but also to support formulation, the manufacturing process, or even manufacturing site changes [9]. For the 3D printed formulations, the number of BE studies required might grow proportionally with the number of formulations/new strengths and new equipment, which might lead to significant cost and patient burden. An alternative solution which would help to reduce the burden is replacing some of the clinical BE studies with PK endpoints by virtual bioequivalence (VBE) studies using physiologically based pharmacokinetic (PBPK) modeling. PBPK modeling is an approach which allows us to predict drug exposure in plasma or target organs while accounting for the differences in patient anatomy, physiology, and demography [10]. In addition to patient-related parameters, it can also estimate the influence of formulation differences on the endpoints of interest. There are a number of examples when PBPK was used in supporting the regulatory decisions, including dose selection and optimization for the newly approved drug, dose adjustment in special populations, and drug–drug interaction studies [11,12,13]. The number of studies where PBPK was used for biopharmaceutical purposes and VBE studies has also grown recently [14,15,16]. In 2019, the FDA approved the first abbreviated new drug application where PBPK was used to support the assessment of the BE of a generic diclofenac topical gel product [17]. The detailed description of this case is provided in [18]. In addition, the FDA encourages applicants to use virtual BE assessment, which not only reduces the cost and patient burden, but also allows the investigation of the sources of variability in the drug’s PK or estimates the risk of formulation changes [19]. The aim of our research was to run a virtual bioequivalence study—and to test its boundaries—for the newly developed 3D printed modified release tablets of ropinirole (test products), compared to the reference product Polpix.

## 2. Materials and Methods

Poly(ethylene glycol) diacrylate (PEGDA), diphenyl(2,4,6-trimethylbenzoyl)phosphine oxide (DPPO), and sodium alginate (AlgNa) were obtained from Sigma-Aldrich, Steinheim, Germany. Ropinirole hydrochloride (RH) was obtained from Pol-Nil, Warsaw, Poland. All other materials were at the analytical grade.

### 2.1. 3D Printing and Dissolution Testing

The 3D printed, oblong tablets (8 mm wide, 16 mm long, and 3 mm height) containing ropinirole hydrochloride (RH) were formulated as 24 h release tablets. For the study’s purpose, twenty-seven formulations were prepared employing a 3^3^ factorial design. Studied factors were PEGDA type (the number average molar mass (M_n_) 700, 575, 250 g/mol), RH concentration (0.5%; 1%; 2%), and AlgNa concentration (0%, 10%, 25%). The formulations were printed using a digital light processing (DLP) Sonik mini 8K printer (Phrozen, Taiwan). For the virtual bioequivalence study, two formulations (F10 and F27) differing in content of AlgNa were chosen. The tablets were printed on the base of PEGDA 700; the ropinirole hydrochloride content in both cases was 2%.

Drug dissolution profiles (*n* = 3 for test formulations, *n* = 7 for the reference formulation) were obtained according to USP Ropinirole Extended-Release Tablets test 1 protocol by USP II apparatus. Tablets in sinkers were placed in 500 mL of citric acid buffer pH = 4.0 for 24 h. The paddle speed of the USP II was fixed at 100 rpm, and the tests were conducted at 37 ± 0.5 °C. Samples (1 mL) were withdrawn at 1, 2, 4, 6, 8, 12, 16, 20, and 24 h time points. Dissolution studies were performed under the same conditions for the commercially available, generic, prolonged release, RH-containing drug, Polpix. RH was assayed using the HPLC Nexera-I LC-2040C 3D Plus system with a PDA detector at 250 nm, operated with LabSolutions DB v.6.82 (Shimadzu, Tokyo, Japan). The HPLC system had a Kinetex^®^ XB-C18 column (particle size 2.6 μm, 100 Å pore size, 3 × 50 mm, Phenomenex, Torrance, CA, USA). The mobile phase was composed of acetate buffer pH = 2.5, acetonitrile, and methanol (800:140:60, *v*/*v*/*v*). It was prefiltered through an OlimPeak^TM^ 0.2 μm hydrophilic PTFE filter (Teknokroma, Barcelona, Spain) and degassed on an ultrasonic degasser. Injections of 8 μL were made. The mixture of water and methanol (50:50, *v*/*v*) was used as a needle rinse solvent. The HPLC oven and PDA detector temperatures were 35 and 40 °C, respectively. To select the 3D printed formulations for the virtual bioequivalence study, the similarity factor (*f*_2_) was used. The similarity factor is a model-independent approach used to calculate the similarity in the dissolution between the two curves of test and a reference formulation. The method is described in the FDA guideline on dissolution testing [20], and *f*_2_ were calculated using the following formula:

(1) $$f_{2}=50\times \log\left[{\left(1+1/n\sum_{t=1}^{n}{{(R}_{t}-T_{t})}^{2}\right)}^{-0.5}\times 100\right]$$

where log is the logarithm to base 10, *n* is the number of time points, Ʃ is the summation over all time points, $R_{t}$ is the dissolution value (%) of the reference product at time *t*, and $T_{t}$ is the dissolution value (%) of the test product at time *t*.

Two profiles were considered similar if the *f*_2_ value was between 50 and 100. Formulation 10 (F10) and Formulation 27 (F27) were considered the most similar in terms of dissolution.

The dissolution profiles of Polpix (REF), F10, and F27 were used as inputs for the PBPK model and were described using Weibull function:

(2) $$F_{diss}=F_{max}\left(1-e^{-{\left(\frac{t-lag}{\alpha }\right)}^{\beta }}\right)$$

where $F_{diss}$ is the fraction of the drug substance dissolved at time *t* (%), $F_{\max}$ is the maximum fraction of the drug substance dissolved (%), *lag*—is the lag time between the beginning of the dissolution study and actual dissolution, and α and β are Weibull scale and shape parameters for the dissolution rate, respectively. A coefficient of variation (CV) of 10% was assigned to each Weibull parameter to cover the interindividual variability for an in vivo dissolution.

It is worth noting that because ropinirole is a BCS class 1 compound, it was assumed that neither dissolution nor permeability were limiting factors in the drug absorption, and it is only the release from the formulation which controlled the absorption. Ropinirole as a BCS class 1 compound does not exert a pH-dependent dissolution. In our modeling and simulation study for the prolonged released formulation, we indirectly proved that a quality control dissolution method was sufficient to describe the drug behavior in the human gastro-intestinal tract (GIT) for the subsequent prediction of concentration-time profiles, e.g., that in vitro dissolution was representative of in vivo dissolution. The PBPK model, together with quality control dissolution data, was able to predict plasma exposure both in healthy volunteers and in Parkinson’s disease patients. The 3D printed formulations were tested in the same conditions as the reference formulation.

### 2.2. PBPK Model Building, Verification, and Application

Ropinirole is an orally administered dopamine receptor agonist indicated in Parkinson’s disease (PD) and Restless Legs Syndrome (RLS) for the management of motor symptoms [21]. The drug is well-soluble and permeable, and predominantly metabolized by CYP1A2 and CYP3A4 enzymes in the liver [22,23]. The ropinirole PBPK model was developed and verified in a Simcyp Simulator for the immediate release formulation, as described in [24]. In summary, the middle-out approach was used in the model building. The hepatic metabolic pathways for ropinirole were mechanistically incorporated into the model using an in vitro–in vivo extrapolation (IVIVE) procedure. The enzyme kinetic parameters were derived from the study of Bloomer et al., 1997 [22], whereas fraction unbound in incubation media was optimized using the clinical dataset and parameter estimation tool in Simcyp. The volume of distribution was predicted using Sawada et al. 1984, an equation based on tissue-to-plasma partition coefficients predicted by the Rodgers and Rowland model [25,26]. After gaining confidence in distribution and elimination, the absorption model was extended to an Advanced Dissolution, Absorption, and Metabolism (ADAM) model to describe drug absorption from the prolonged release formulation [27]. The model was validated using concentration-time profiles for healthy volunteers (Sim-Healthy Volunteers virtual population), and subsequently for Parkinson’s disease patients (Sim-North European virtual population). Each of 10 virtual trials included up to 50 subjects (up to 500 subjects in total; the number of subjects varied depending on the clinical trial design). Regarding the model validation criteria, there are no formally recognized acceptance criteria established by the regulatory bodies for the model quality assessment [19]; therefore, commonly accepted 2-fold criteria for simulated vs observed Cmax (maximum plasma concentration) and AUC (area under the concentration-time curve) were used [28,29,30]. Visual predictive checks were also used to make sure there was no systematic misprediction in any of the PK components, e.g., absorption, distribution, or elimination. Based on these criteria, the above cited models successfully predicted ropinirole plasma exposure in healthy and Parkinson’s disease patients after single and multiple dose administration of immediate and prolonged release formulations.

In this study, we also verified the quantitative contribution of liver enzymes to the ropinirole elimination by simulating the interaction study with CYP1A2 inhibitor ciprofloxacin, using the verified inhibitor compound file from the Simcyp library [31].

The aim of this paper is to describe the next step following model verification: its application to analyze bioequivalence of the newly developed 3D printed ropinirole tablets.

### 2.3. VBE Study Power Calculations

The power analysis was performed to define a sufficient patient number to cover the safe space analysis. The calculations were conducted using the Simcyp build-in module where the study power is defined as the probability of detecting a difference between two populations for a particular parameter given that there is a true difference between these populations. The parameters of interest were Cmax, AUC_0–t_, and AUC_0–inf_. It was assumed that the listed parameters follow normal distributions, for example, if the Cmax for Population 1 is *x* and the same parameter for Population 2 is *y*, then:

(3) $$x\sim N({\mu_{1},\sigma_{1}}^{2})$$

(4) $$y\sim N({\mu_{2}{,\sigma }_{2}}^{2})$$

where ${\mu_{1}\;and\;\sigma_{1}}^{2}$ are the mean and variance for Population 1, and ${\mu_{2}\;and\;\sigma_{2}}^{2}$ are the mean and variance for Population 2. The null hypothesis tested was that Population 2 is equal to Population 1 in terms of distribution., e.g., it has the same mean and variance. The mean and variance of the parameter for each population was calculated based on the number of individuals in the trial design of the module.

According to the central limit theorem, if a sample with the size of $n_{1}$ is selected from Population 1 and a sample with the size of $n_{2}$ is selected from Population 2, then the normal distribution for sample means $\bar{x}\;and\;\bar{y}$ is:

(5) $$\bar{x}\sim N(\mu_{1},\;\frac{{\sigma_{1}}^{2}}{n_{1}})$$

(6) $$\bar{y}\sim N(\mu_{2},\;\frac{{\sigma_{2}}^{2}}{n_{2}})$$

To calculate the power, the critical value was calculated for Population 1 at the significance level of 0.05. The critical value defines whether the null hypothesis is rejected. The power was then calculated using the critical value and the distribution for Population 2. The number of subjects tested in the power analysis ranged from 8 to 200. The subjects for Population 1 and Population 2 were selected by the simulator from the Sim-Healthy Volunteers library.

### 2.4. Virtual Bioequivalence Trial Design

For virtual BE assessment, the Simcyp VBE module, which is part of the Simcyp Simulator (V22), was utilized [32]. The VBE tool allows simulation of various VBE studies for drugs applied via various routes of administration. Both crossover and parallel trial designs can be simulated, and the custom BE design option enables users to define complex study scenarios. Depending upon the crossover study design (standard crossover trial design or partial or full replicate crossover trial design), each simulated individual, randomly picked from a desired population, receives both reference and test formulations. Simulations are performed for each individual in the different periods, with both intra- and inter-individual variability defined for a selection of desired model parameters. The inter-individual variability is applied based on the correlated Monte Carlo simulation in a similar way as in the main simulator [33]. As far as application of the inter-occasion variability is available, its parametrization is still challenging due to limited data available in the publicly available sources. There are methods used to overcome this challenge, for example, a statistical (or population pharmacokinetics) based approach [34]. For our purposes, the parallel study design was used, which is considered a worst-case scenario in term of influence of variability on the obtained results for the VBE assessment. It assumes that inter-occasion variability is equal to inter-individual variability. The typical pharmacokinetic endpoints, Cmax, AUC_0–t_, and AUC_0–inf_, were tested in terms of geometric mean ratios of parameters for test and reference formulations. The formulations were assumed to be bioequivalent if the 90% confidence intervals of the test/reference geometric mean for the listed PK parameters fall within the bioequivalence limits of 80–125% [35]. Finally, the number of virtual study replicates was 1, while 10 studies were simulated only for diagnostic purposes for one group size for the pair REF-F27, which was assumed to be bioequivalent. Each of the 3D printed formulations were compared to reference formulation (REF), and between themselves, the tested pairs were F10:REF, F27:REF, and F10:F27.

## 3. Results

### 3.1. Dissolution Profiles

**Figure 1:**
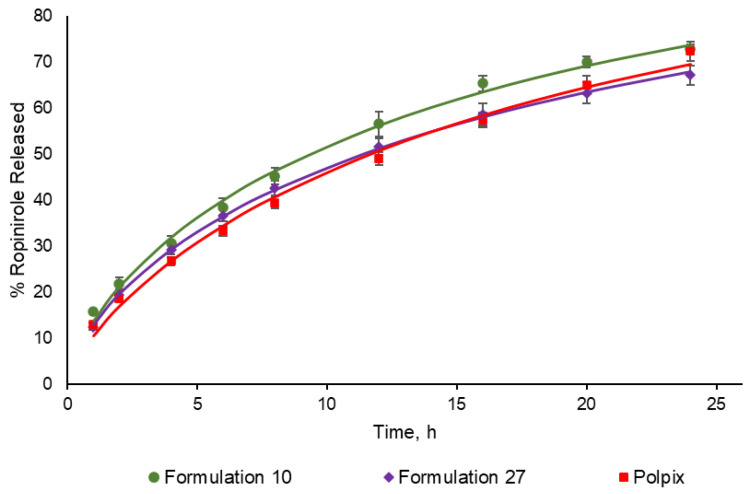
Mean dissolution profiles (*n* = 3 for F10 and F27, *n* = 7 for Polpix, ±SD) for 3D printed tested formulations and reference product Polpix. Points indicate data observed at specific timepoints while lines indicate Weibull-fitted profiles.

For all tested formulations, the f2 parameter was calculated to compare their similarity to the reference formulation (Polpix) in terms of dissolution profile. The composition of the chosen 3D printed formulations together with similarity factors are presented in Table 1:

The mean 24 h dissolution profiles for F27, F10, and the reference product Polpix are presented in Figure 1.

### 3.2. Simulation of Ropinirole Interaction with Ciprofloxacin

**Figure 2:**
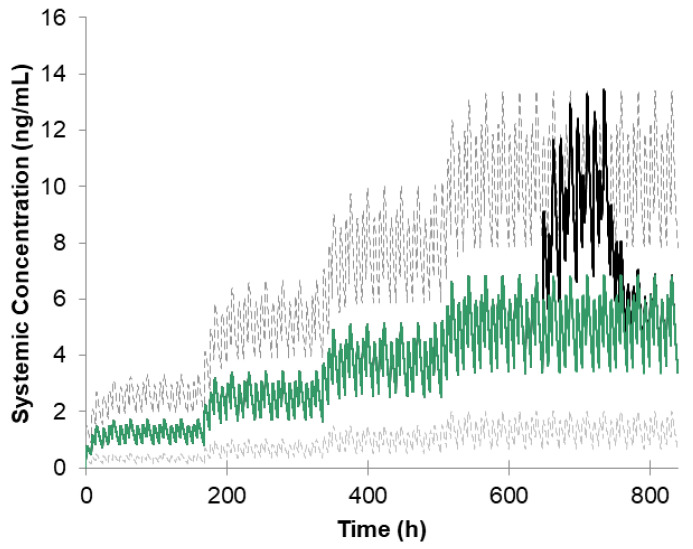
Simulated plasma concentration-time profile for ropinirole given at a dose of 0.5–2 mg TID (green solid line) together with ciprofloxacin 500 mg BID (black solid line) given on Day 28 of the virtual trial. Gray dashed lines represent the fifth and ninety-fifth percentiles.

The simulation of ropinirole’s CYP-mediated interaction with ciprofloxacin was performed according to the trial design described in the Scientific Result Summary of Study 101468/102 [31]. Ropinirole was administered as an immediate release formulation to 12 Parkinson’s disease patients during 35 days in a dose from 0.5 mg to 2 mg TID. The dose was increased every week by 0.5 mg and kept at the level of 2 mg during the last week. Ciprofloxacin was given orally 500 mg BID in the last week. The pharmacokinetic points to estimate the level of interaction were measured at steady state. The study was simulated using the ropinirole compound file mentioned earlier, and the ciprofloxacin compound file from the Simcyp Compound File Library. The number of virtual subjects for this additional PBPK simulation was 12 in each of 10 virtual trials (120 subjects in total). The Ki values for ciprofloxacin inhibiting CYP1A2 and CYP3A4 were 2 µM and 1.5 µM, respectively, while values for fraction unbound in incubation media for the same enzymes were 0.92 and 1, respectively (please see Supplementary Materials for all of the PBPK input parameters). The simulated concentration-time profile is shown in Figure 2. The observed profile was not available in the study summary. The comparison of simulated vs observed PK parameters at steady state is provided in Table 2.

### 3.3. Study Power Calculation

**Figure 3:**
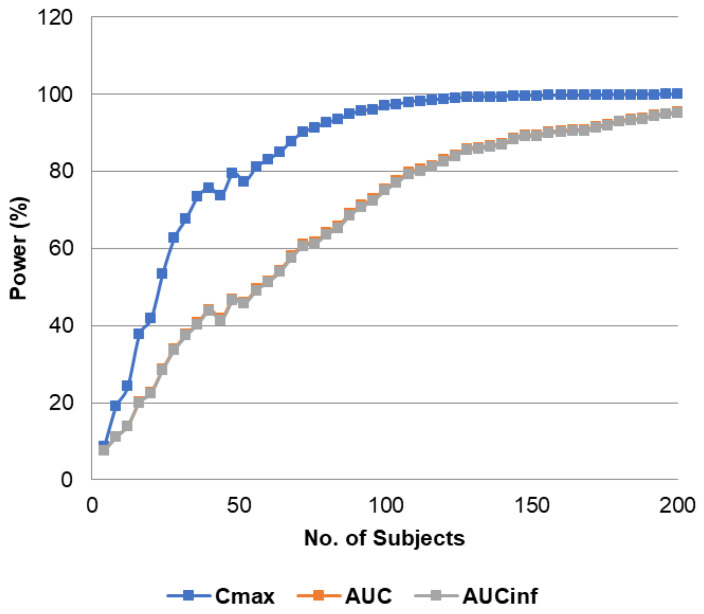
Calculation of study power vs. number of individuals for Cmax, AUC_0–t_, and AUC_0–inf_.

The parallel study power analysis suggested that ~125 individuals will be needed to achieve 80% power for AUC_0–t_ and AUC_0–inf_. The calculation results are presented as a graph in Figure 3.

### 3.4. Virtual Bioequivalence Study

**Figure 4:**
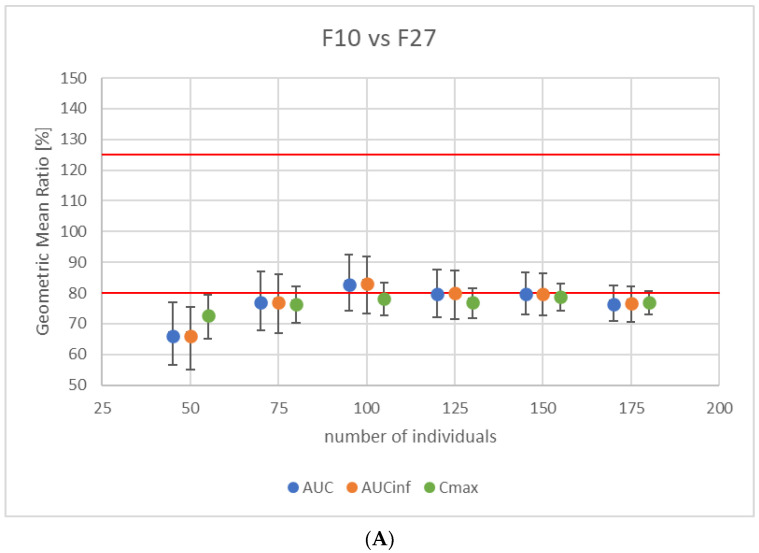

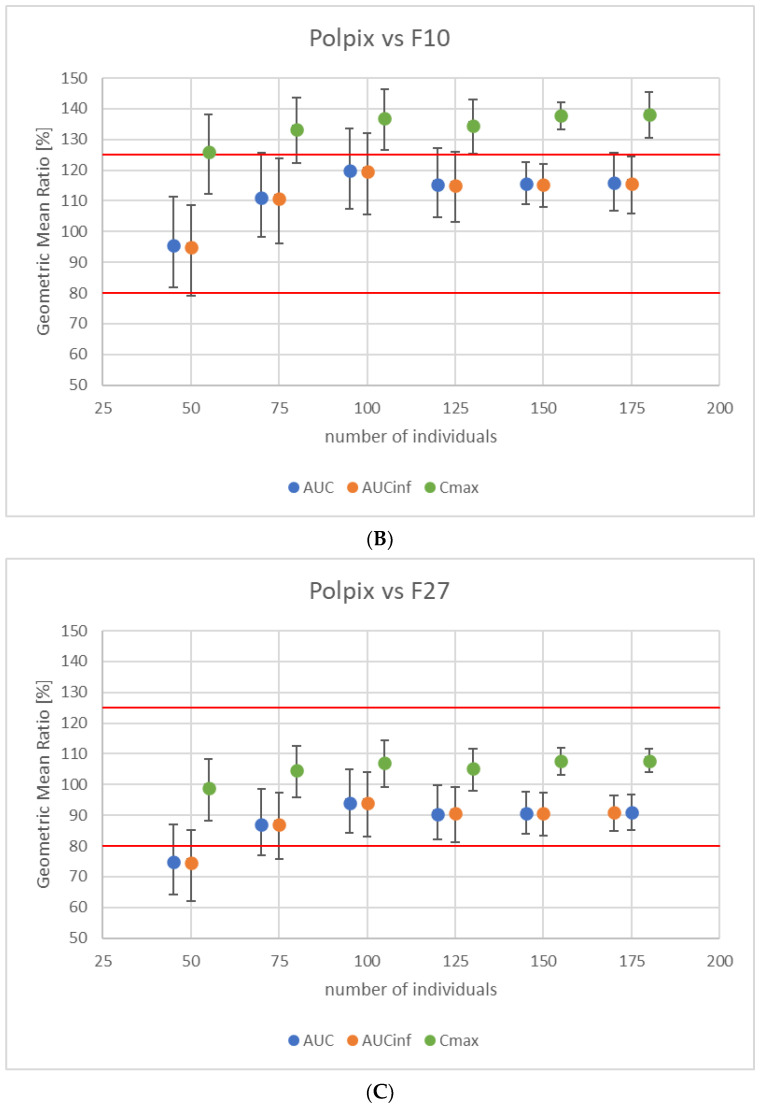
Results of virtual BE studies for three tested formulations: Polpix (REF), F10, and F27 (T). (**A**) F10 vs F27, (**B**) Polpix vs F10, and (**C**) Polpix vs F27. Circles represent T/REF geometric mean ratios and whiskers represent the 90% confidence interval. Red lines represent the bioequivalence acceptance criteria, namely the 80–125% of geometric mean ratio.

Two formulations were included in the VBE analysis, based on the calculated f2 values, namely F10 and F27. The virtual BE studies were run in a parallel study regime as described above. All three formulations were tested in pairs: formulation REF vs F10, REF vs F27, and F10 vs F27. Results of the BE analysis in terms of geometric mean ratios for the indicated pharmacokinetic endpoints are presented below in Figure 4A–C.

**Figure 5:**
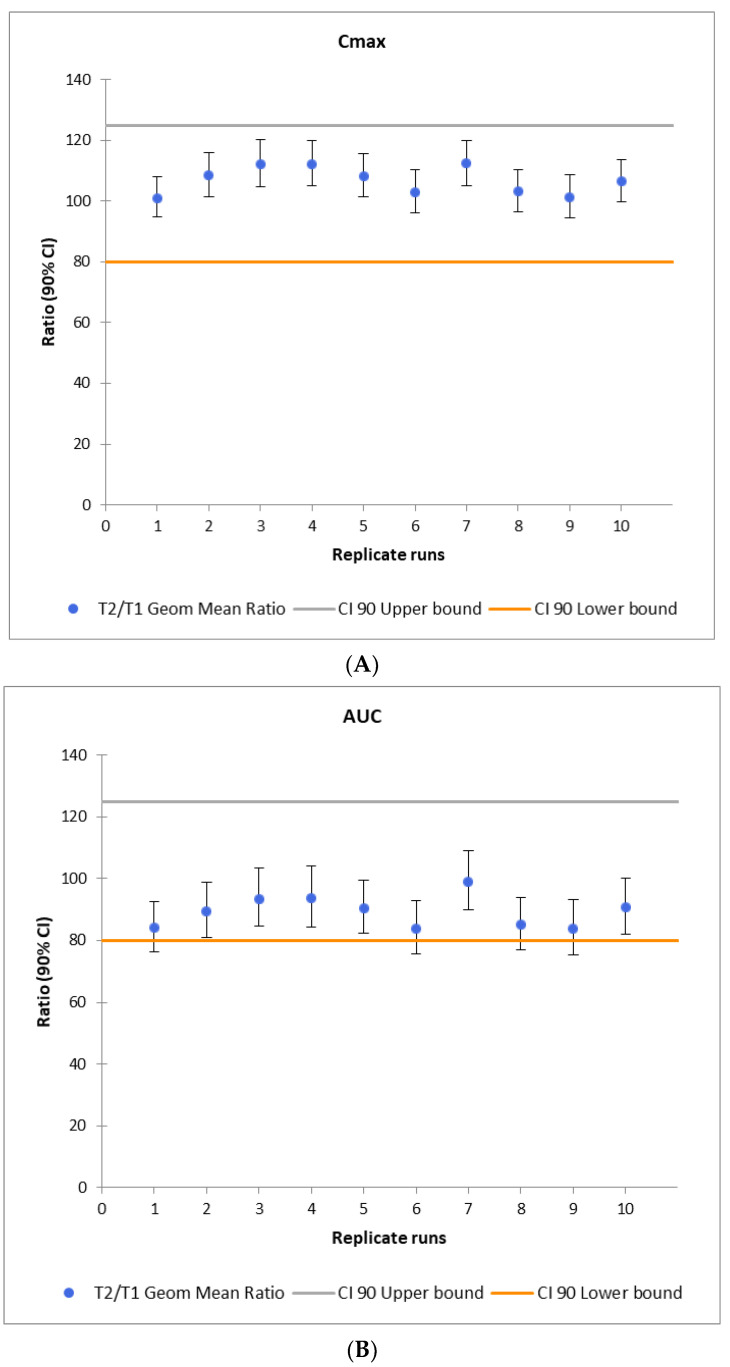

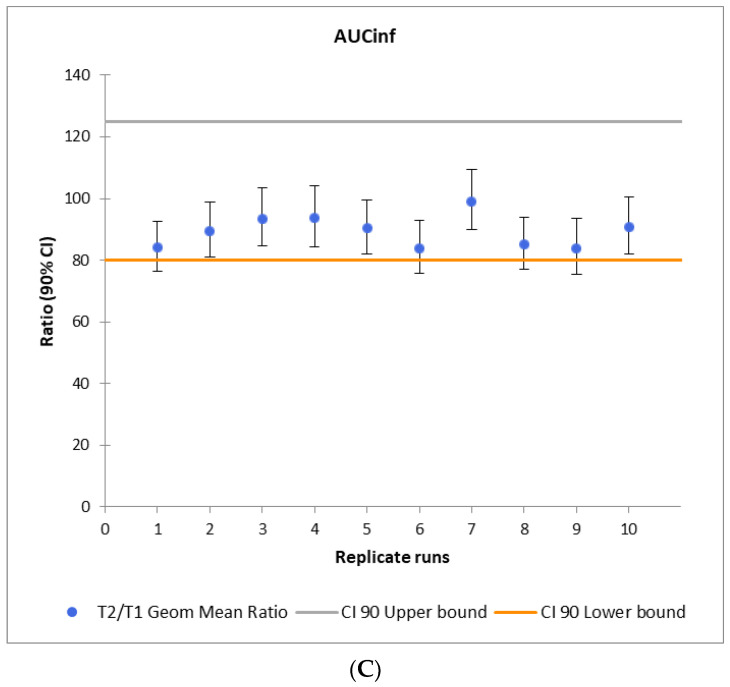
Results of multiple virtual BE studies for Polpix vs. F27, with 125 individuals in each from parallel populations. The pharmacokinetic endpoints include Cmax (**A**); AUC_0–t_ (**B**); AUC_inf_ (**C**). Circles represent T/R geometric means ratios for C_max_, AUC_0–t_, and AUC_inf_, respectively, and whiskers represent the 90% confidence interval.

To test the model sensitivity for the Polpix vs F27 scenario, ten virtual BE studies were run with the same scenario and different seed for each out of ten virtual pairs (test and reference arm) of populations. The results are presented below in Figure 5A–C.

## 4. Discussion

The aim of the study was to assess the ability of using the PBPK based virtual bioequivalence tool to compare 3D printed generic tablets vs the reference drug. Additionally, the combination of 3D printing and in silico prediction tools for estimation of bioequivalence of newly developed formulations is proposed. Ropinirole, BCS class 1 drug, was used as an example. The drug substance for the 3D printed formulation development was chosen based on the biopharmaceutical classification to minimize substance-related confounding factors in dissolution. The PBPK model used in this study was developed and verified earlier in healthy volunteers and Parkinson’s disease patients. As part of this work, we simulated a drug–drug interaction study with the use of CYP1A2 inhibitor ciprofloxacin to further validate the PBPK model. The observed vs simulated DDI ratios for AUC and Cmax were 1.17 and 1.36, respectively, which indicates some level of overprediction of the interaction magnitude; however, it is still well within the 2-fold error which was used as an acceptance criterion.

Because neither dissolution nor permeability are rate-limiting factors in ropinirole absorption, this process was described semi-mechanistically, e.g., assuming that in vitro dissolution profiles are representative of the in vivo dissolution. We would like to emphasize that such an approach partially limits the modeler ability to account for drug/formulation properties and patient physiology interaction, however, it is also worth mentioning that the mechanisms of substance release from 3D printed formulations are different from those in classical manufacturing. Due to this, the mechanistic absorption models need to be revised to be able to account for the key 3D printed formulation parameters in the model. Such release mechanisms and key formulation parameters require more thorough research and understanding with the use of advanced analytical techniques. Nevertheless, we were able to use the tools we had to demonstrate the 3D manufacturing and PBPK combination workflow to predict bioequivalence.

Before the analysis, the study power calculations were performed, where it was shown that to reach the study power of 80%, 125 individuals in a virtual trial were needed. The recommended bioequivalence study design does not differ much among regulatory agencies across the world and is a randomized, single-dose, two-period, crossover study in healthy individuals [35]. However, in our VBE study we used parallel study design considering the lack of data on inter-occasion variability. With that, the worst-case scenario was tested, where inter-occasion variability was assumed to be equal to interindividual variability.

As can be seen in Figure 4, F27 can be considered bioequivalent to Polpix based on the acceptance criteria of 80–125% of geometric mean ratio for all three PK endpoints (Figure 4C). F10 failed to demonstrate bioequivalence in terms of Cmax (Figure 4B). The acceptance criteria were also not met for the comparison of newly printed formulations between themselves (Figure 4A).

While PBPK and VBE can be applied for setting clinically relevant specifications or regulatory decision making, the aim of this work was to estimate the bioequivalence of newly developed 3D printed formulations in the experimental settings. Considering that 3D printing is still a relatively new method in formulation development, the BCS class 1 compound was chosen as a prototype drug enabling focusing on the effect of excipients and printing technology rather than the active substance absorption properties. To be able to model the drug release from 3D printed formulation, more understanding of this process is needed. Currently, the dissolution profiles were used as direct inputs for the model. In our experiments, we did not observe high variability in the in vitro dissolution profiles (please see Figure 1). One of the reasons is a relatively small number of tablets, followed by using the same printing equipment. Nevertheless, in real-life conditions, the variability will highly depend on the equipment, manufacturing process, and applied dissolution methodology. The question of minimizing and controlling the variability is one of the 3D printing challenges. While directly using in vitro dissolution data as in silico model input, one should consider that variability of in vivo dissolution will differ from the in vitro conditions. Considering ropinirole BCS class, we did not expect its in vivo dissolution to be the main source of the inter-subject variability in pharmacokinetics, however such an assumption would be difficult to make for the drugs with permeability or dissolution limitations and more mechanistic absorption models will be needed to be applied [36].

Additionally, while the 3D printing approach is a promising tool in dose personalization, with its main benefit lying in the production of small batches of drug products with individualized doses and release profiles [37], it is also being perceived critically. A big challenge is to ensure the quality of the final 3D printed solid drug formulations, because the destructive analytical characterization methods like in vitro dissolution or disintegration tests, based on many repetitions, are not applicable to small batches produced at the point-of-care.

## 5. Conclusions

Three-dimensional printing is an emerging technology for pharmaceutical applications, and its flexibility, low costs, and the speed of manufacturing formulation open new perspectives for personalization of drug therapy. In this work, we suggest the use of 3D printing in conjunction with PBPK–VBE modeling workflow as a tool to reduce the bioequivalence test burden for the potentially large number of 3D printed formulations with the release and strength tailored for individual patient needs. Three-dimensional printing is not widely applied in pharmaceutical manufacturing; therefore, more analytical studies are needed to understand and model the drug release from the formulation more mechanistically. However, by using the in silico tools and knowledge we have, it is already possible to preliminarily estimate the formulation bioequivalence in experimental settings.

## Figures and Tables

**Table 1:** Composition of 3D printed formulations and calculated similarity factors.

	Formulation 10	Formulation 27
Type of PEGDA	700	700
Sodium Alginate concentration [%]	10	0
RH concentration [%]	2	2
Similarity factor (*f*_2_)	57.44	71.02

**Table 2:** PK parameters from a ropinirole–ciprofloxacin interaction study and their comparison with the simulated results.

Parameter	Ropinirole Alone, Mean	Ropinirole + Ciprofloxacin, Mean	Geometric Mean Ratio	Observed/ Simulated Ratio
AUC_0–6_ ng.h/mL observed	30.2	57	1.84	1.17
AUC_0–6_ ng.h/mL simulated	31.40	66.85	2.16	
Cmax (ng/mL) observed	7.86	12.67	1.6	1.36
Cmax (ng/mL) simulated	5.98	12.70	2.17	

## Data Availability

The original contributions presented in the study are included in the article/Supplementary Material, further inquiries can be directed to the corresponding author/s.

## Supplementary Materials

The following supporting information can be downloaded at: https://www.mdpi.com/article/10.3390/pharmaceutics16020259/s1. The file represents Simcyp simulation output (V22) for ropinirole-ciprofloxacin interaction study.

## References

[1] Chetty M., Rose R.H., Abduljalil K., Patel N., Lu G., Cain T., Jamei M., Rostami-Hodjegan A. (2014). Applications of linking PBPK and PD models to predict the impact of genotypic variability, formulation differences, differences in target binding capacity and target site drug concentrations on drug responses and variability. Front. Pharmacol..

[2] De Sousa Mendes M., Chetty M. (2019). Are Standard Doses of Renally-Excreted Antiretrovirals in Older Patients Appropriate: A PBPK Study Comparing Exposures in the Elderly Population with Those in Renal Impairment. Drugs R D.

[3] Xie H.-G., Frueh F.W. (2005). Pharmacogenomics steps toward personalized medicine. Pers. Med..

[4] Gonzalez D., Rao G.G., Bailey S.C., Brouwer K.L.R., Cao Y., Crona D.J., Kashuba A.D.M., Lee C.R., Morbitzer K., Patterson J.H. (2017). Precision Dosing: Public Health Need, Proposed Framework, and Anticipated Impact. Clin. Transl. Sci..

[5] El Aita I., Breitkreutz J., Quodbach J. (2019). On-demand manufacturing of immediate release levetiracetam tablets using pressure-assisted microsyringe printing. Eur. J. Pharm. Biopharm..

[6] Skowyra J., Pietrzak K., Alhnan M.A. (2015). Fabrication of extended-release patient-tailored prednisolone tablets via fused deposition modelling (FDM) 3D printing. Eur. J. Pharm. Sci..

[7] Lafeber I., Tichem J.M., Ouwerkerk N., van Unen A.D., van Uitert J.J.D., Bijleveld-Olierook H.C.M., Kweekel D.M., Zaal W.M., Le Brun P.P.H., Guchelaar H.J. (2021). 3D printed furosemide and sildenafil tablets: Innovative production and quality control. Int. J. Pharm..

[8] Karalia D., Siamidi A., Karalis V., Vlachou M. (2021). 3D-Printed Oral Dosage Forms: Mechanical Properties, Computational Approaches and Applications. Pharmaceutics.

[9] FDA (2022). Guidance for Industry Bioavailability Studies Submitted in NDAs or INDs—General Considerations, U.S. Department of Health and Human Services Food and Drug Administration Center for Drug Evaluation and Research (CDER). https://www.fda.gov/regulatory-information/search-fda-guidance-documents/bioavailability-studies-submitted-ndas-or-inds-general-considerations.

[10] Rowland M., Peck C., Tucker G. (2011). Physiologically-based pharmacokinetics in drug development and regulatory science. Annu. Rev. Pharmacol. Toxicol..

[11] Templeton I.E., Jones N.S., Musib L. (2018). Pediatric Dose Selection and Utility of PBPK in Determining Dose. AAPS J..

[12] Heimbach T., Chen Y., Chen J., Dixit V., Parrott N., Peters S.A., Poggesi I., Sharma P., Snoeys J., Shebley M. (2021). Physiologically-Based Pharmacokinetic Modeling in Renal and Hepatic Impairment Populations: A Pharmaceutical Industry Perspective. Clin. Pharmacol. Ther..

[13] Ganti A., Yu S., Sharpnack D., Ingalla E., De Bruyn T. (2023). Physiologically-based pharmacokinetic/pharmacodynamic modeling to predict tumor growth inhibition and the efficacious dose of selective estrogen receptor degraders in humans. Biopharm. Drug Dispos..

[14] Doki K., Darwich A.S., Patel N., Rostami-Hodjegan A. (2017). Virtual bioequivalence for achlorhydric subjects: The use of PBPK modelling to assess the formulation-dependent effect of achlorhydria. Eur. J. Pharm. Sci..

[15] Laisney M., Heimbach T., Mueller-Zsigmondy M., Blumenstein L., Costa R., Ji Y. (2022). Physiologically Based Biopharmaceutics Modeling to Demonstrate Virtual Bioequivalence and Bioequivalence Safe-space for Ribociclib which has Permeation Rate-controlled Absorption. J. Pharm. Sci..

[16] Loisios-Konstantinidis I., Cristofoletti R., Fotaki N., Turner D.B., Dressman J. (2020). Establishing virtual bioequivalence and clinically relevant specifications using in vitro biorelevant dissolution testing and physiologically-based population pharmacokinetic modeling. case example: Naproxen. Eur. J. Pharm. Sci..

[17] FY2019 GDUFA Research Report: Locally Acting Physiologically Based Pharmacokinetic Modeling: Summary of FY2019 Activities. https://www.fda.gov/media/135187/download#page=35.

[18] Tsakalozou E., Babiskin A., Zhao L. (2021). Physiologically-based pharmacokinetic modeling to support bioequivalence and approval of generic products: A case for diclofenac sodium topical gel, 1. CPT Pharmacomet. Syst. Pharmacol..

[19] Tsakalozou E., Alam K., Babiskin A., Zhao L. (2022). Physiologically-Based Pharmacokinetic Modeling to Support Determination of Bioequivalence for Dermatological Drug Products: Scientific and Regulatory Considerations. Clin. Pharmacol. Ther..

[20] FDA Guidance for Industry SUPAC-MR: Modified Release Solid Oral Dosage Forms Scale-Up and Postapproval Changes: Chemistry, Manufacturing, and Controls; In Vitro Dissolution Testing and In Vivo Bioequivalence Documentation 1997. https://www.fda.gov/regulatory-information/search-fda-guidance-documents/supac-mr-modified-release-solid-oral-dosage-forms-scale-and-postapproval-changes-chemistry.

[21] Ferraiolo M., Hermans E. (2023). The complex molecular pharmacology of the dopamine D2 receptor: Implications for pramipexole, ropinirole, and rotigotine. Pharmacol. Ther..

[22] Bloomer J.C., Clarke S.E., Chenery R.J. (1997). In vitro identification of the P450 enzymes responsible for the metabolism of ropinirole. Drug Metab. Dispos..

[23] Iwasaki S., Yamamoto S., Sano N., Tohyama K., Kosugi Y., Furuta A., Hamada T., Igari T., Fujioka Y., Hirabayashi H. (2019). Direct Drug Delivery of Low-Permeable Compounds to the Central Nervous System Via Intranasal Administration in Rats and Monkeys. Pharm. Res..

[24] Shuklinova O., Dorożyński P., Kulinowski P., Bielecka Z., Wiśniowska B., Polak S. (2021). Development of physiologically based pharmacokinetic model for the immediate release of ropinirole tablets. Acta Pol. Pharm. Drug Res..

[25] Sawada Y., Hanano M., Sugiyama Y., Harashima H., Iga T. (1984). Prediction of the volumes of distribution of basic drugs in humans based on data from animals. J. Pharmacokinet. Biopharm..

[26] Rodgers T., Leahy D., Rowland M. (2005). Physiologically based pharmacokinetic modeling 1: Predicting the tissue distribution of moderate-to-strong bases. J. Pharm. Sci..

[27] Shuklinova O., Dorożyński P., Kulinowski P., Polak S. (2022). Quality control dissolution data is biopredictive for a modified release ropinirole formulation: Virtual experiment with the use of re-developed and verified PBPK model. Pharmaceutics.

[28] Abduljalil K., Cain T., Humphries H., Rostami-Hodjegan A. (2014). Deciding on success criteria for predictability of pharmacokinetic parameters from in vitro studies: An analysis based on in vivo observations. Drug Metab. Dispos..

[29] Kuemmel C., Yang Y., Zhang X., Florian J., Zhu H., Tegenge M., Huang S.-M., Wang Y., Morrison T., Zineh I. (2020). Consideration of a Credibility Assessment Framework in Model-Informed Drug Development: Potential Application to Physiologically-Based Pharmacokinetic Modeling and Simulation. CPT Pharmacomet. Syst. Pharmacol..

[30] Shebley M., Sandhu P., Emami Riedmaier A., Jamei M., Narayanan R., Patel A., Peters S.A., Reddy V.P., Zheng M., de Zwart L. (2018). Physiologically Based Pharmacokinetic Model Qualification and Reporting Procedures for Regulatory Submissions: A Consortium Perspective. Clin. Pharmacol. Ther..

[31] GSK—A Study to Investigate the Effect of Repeated Oral Doses of Ciprofloxacin on Steady State Ropinirole Pharmacokinetics in Parkinsonian Patients. https://www.gsk-studyregister.com/en/trial-details/?id=101468/102.

[32] Clarke J.F., Thakur K., Polak S. (2022). A mechanistic physiologically based model to assess the effect of study design and modified physiology on formulation safe space for virtual bioequivalence of dermatological drug products. Front. Pharmacol..

[33] Jamei M., Marciniak S., Feng K., Barnett A., Tucker G., Rostami-Hodjegan A. (2009). The Simcyp population-based ADME simulator. Exp. Opin. Drug Metab. Toxicol..

[34] Danielak D., Paszkowska J., Staniszewska M., Garbacz G., Terlecka A., Kubiak B., Romański M. (2023). Conjunction of semi-mechanistic in vitro-in vivo modeling and population pharmacokinetics as a tool for virtual bioequivalence analysis—A case study for a BCS class II drug. Eur. J. Pharm. Biopharm..

[35] Davit B., Braddy A.C., Conner D.P., Yu L.X. (2013). International Guidelines for Bioequivalence of Systemically Available Orally Administered Generic Drug Products: A Survey of Similarities and Differences. AAPS J..

[36] Wyszogrodzka-Gaweł G., Shuklinova O., Lisowski B., Wiśniowska B., Polak S. (2023). 3D printing combined with biopredictive dissolution and PBPK/PD modeling optimization and personalization of pharmacotherapy: Are we there yet?. Drug Discov. Today.

[37] Trenfield S.J., Awad A., Goyanes A., Gaisford S., Basit A.W. (2018). 3D Printing Pharmaceuticals: Drug Development to Frontline Care. Trends Pharmacol. Sci..

